# Prediction of Nociceptive Responses during Sedation by Linear and Non-Linear Measures of EEG Signals in High Frequencies

**DOI:** 10.1371/journal.pone.0123464

**Published:** 2015-04-22

**Authors:** Umberto Melia, Montserrat Vallverdú, Xavier Borrat, Jose Fernando Valencia, Mathieu Jospin, Erik Weber Jensen, Pedro Gambus, Pere Caminal

**Affiliations:** 1 Dept. ESAII, Universitat Politècnica de Catalunya, Pau Gargallo 5, 08028, Barcelona, Spain; 2 Centre for Biomedical Engineering Research, Pau Gargallo 5, 08028, Barcelona, Spain; 3 CIBER-BBN, Pau Gargallo 5, 08028, Barcelona, Spain; 4 Systems Pharmacology Effect Control & Modeling (SPEC-M) Research Group, Anesthesiology Department, Hospital CLINIC de Barcelona, Barcelona, Spain; 5 Neuroimmunology Research Program Institut de Investigacions Biomèdiques August Pi Sunyer (IDIBAPS), Barcelona, Spain; 6 University of San Buenaventura, Dept. Electronic Engineering, Cali, Colombia; 7 R&D Dept. Quantium Medical SL, Mataró, Spain; 8 Department of Anesthesia and Perioperative Medicine, University of California San Francisco, San Francisco, California, United States of America; Universiteit Gent, BELGIUM

## Abstract

The level of sedation in patients undergoing medical procedures evolves continuously, affected by the interaction between the effect of the anesthetic and analgesic agents and the pain stimuli. The monitors of depth of anesthesia, based on the analysis of the electroencephalogram (EEG), have been progressively introduced into the daily practice to provide additional information about the state of the patient. However, the quantification of analgesia still remains an open problem. The purpose of this work is to improve the prediction of nociceptive responses with linear and non-linear measures calculated from EEG signal filtered in frequency bands higher than the traditional bands. Power spectral density and auto-mutual information function was applied in order to predict the presence or absence of the nociceptive responses to different stimuli during sedation in endoscopy procedure. The proposed measures exhibit better performances than the bispectral index (BIS). Values of prediction probability of P_k_ above 0.75 and percentages of sensitivity and specificity above 70% were achieved combining EEG measures from the traditional frequency bands and higher frequency bands.

## Introduction

To determine appropriate requirements for administration, monitoring and control of sedation and / or analgesia in invasive medical procedures is necessary in order to minimize the impact of the aggression in the patient and the implications on the outcome of the process. Hypnotic drugs (intravenous or inhaled) are used in order to achieve an accurate level of hypnosis, while fundamentally strong opioids are used in order to achieve the desired level of analgesia. The proper implementation of proposed model to monitor the anesthetic state is based on a quantification of the pharmacological effects for reaching a perfect set respect to the requirements of each patient. This involves the development of models connected directly in real-time with physiological variables of the patient. For several years, various methods have been developed for the noninvasive assessment of the level of consciousness during general anesthesia [1–6]. Since the main action of anesthetic agents occurs in the brain, a reasonable choice is to monitor the electroencephalographic signal (EEG). Changes on the EEG signal are directly related to biochemical variations of a drug induced in the brain and the effects on individual behavior. For this reason, different EEG monitors have been developed [7–12]. Analysis of the Bispectrum of the EEG signal, entropy analysis, and auditory evoked potentials extracted from the EEG or automated neurofuzzy inference systems are some of the methods applied to the complex EEG signal to design clinically relevant indicators of hypnotic effect.

However, it has not been possible to develop a system capable of quantifying analgesia. The most used methods [13–16] include hemodynamic response, analysis of electrocardiographic waveforms variability, degree of respiratory sinus arrhythmia, plethysmographic response, pulse wave, heart rate variability and skin conductance. None of them has proven to be clinically useful methods because they are influenced by the response of the autonomic nervous system (ANS) and they are sensitive to other disturbances, such as changes in blood pressure or heart rate due to patient's baseline condition (hypertension, arrhythmias of diverse etiology), sympathomimetic drug delivery or unpredictable situations such as perioperative bleeding. Recently, two studies based on time-frequency representation [17] and auto-mutual information function [18] demonstrated that changes associated to EEG spectrum and EEG complexity in the traditional bands permitted to improve the prediction of the Ramsay sedation scale (RSS) and the response to tube insertion during endoscopy procedure. However, the discrimination between deep sedation level with no response to any stimulation (RSS = 6) and sluggish response to painful stimulation (RSS = 5) still remains an open problem. Some studies indicated that the EEG-based monitors cannot reliably distinguish between light sedation and deep sedation, as these are designed to measure levels of general anesthesia that handle very different levels of hypnosis to those used in sedation procedures. Therefore still remains the need for an objective measurement to quantify the level of sedation in patients undergoing invasive procedures. Additionally, during sedation procedures patients develop a greater degree of muscular activity compared to patients undergoing general anesthesia procedures [19]. A biopotential signal measured from the forehead of a patient includes a significant EMG component, which is created by muscle activity. The EMG signal has a wide noise-like spectrum and during anesthesia typically dominates at frequencies higher than 30 Hz. Sudden appearance of EMG signal data often indicates that the patient is responding to some external stimulus, such as a painful stimulus, due to some surgical event. Such a response may result if the level of analgesia is insufficient. If stimulation continues and no additional analgesic drugs are administered, it is highly likely that the level of hypnosis starts to reduce. Thus, EMG can help to provide a rapid indication of imminent arousal [9].

In this work, we assume that the prediction of the responses to pain stimulation during endoscopy procedure can be improved by using measures calculated in the recorded EEG taking into account also the EMG frequency bands derived from scalp and facial muscles. In this sense, it might be possible to associate an increased activity in the facial muscles with a greater possibility of pain, obtaining better prediction of responsive states. To achieve our goal, linear and non-linear measures were calculated on the EEG signal in the traditional bands *δ*, *θ*, *α*, *β* and in higher frequency bands (*HF*: 60–95 Hz and *VHF*: 105–145 Hz). Several measures based on power spectral density and auto-mutual information function were defined in order to evaluate the prediction of responding to the application of painful stimuli such as nail bed compression or endoscopy tube insertion.

## Materials and Methods

### Database

The analyzed database belongs to the Department of Anesthesiology, Hospital Clínic of Barcelona (Spain). This database contains data recorded from 378 patients (mean age 63±23 years, 247 men) undergoing ultrasonographic endoscopy of the upper gastrointestinal tract under sedation and analgesia with propofol and remifentanil. All the patients belong to 1–3 ASA classification. Patients with altered central nervous system, medicated with analgesics or drugs with central effects on the perception of pain, from moderate to severe cardiomyopathy, neuropathy or hepatopathy that needed control during the anesthetic process were not included in the database. The study received approval from the Ethics Committee of Hospital Clinic de Barcelona and all the patients signed informed consent.

For each patient, the following information is available: predicted effect site concentrations of propofol (*Ce*_*Prop*_) and remifentanil (*Ce*_*Remi*_); bispectral Index (*BIS*) and electroencephalogram (EEG) signal. The observed categorical responses after applied nociceptive stimuli include the evaluation of the RSS (see Table 1) [20] after nail bed compression and the presence of gag reflex during endoscopy tube insertion (GAG). Specifically, RSS 2, 3, 4 and 5 corresponds to a patient who responded with purposeful movement after nail bed compression while patients in the RSS 6 category did not respond. GAG corresponds to a positive nausea reflex during endoscopy tube insertion, a nociceptive stimulus as well. The RSS score was evaluated at random times during the procedure in order to avoid those factors correlated with time, which could confound the results of the RSS measurements. The whole database contains annotated RSS scores from 2 to 6.

**Table 1:** Ramsay sedation scale.

Score	Response
1	Anxious and/or restless
2	Cooperative, orientated and calm
3	Responding to instructions
4	Brisk response to stimulus
5	Sluggish response to stimulus
6	No response to stimulus

The EEG was recorded with a sampling frequency of 900 Hz, with a resolution of 16 bits and a recording time of about 60 min using AEP monitor/2 (Danmeter, Odense, Denmark). A 3-electrode montage was used: middle forehead (+), malar bone (-), and left forehead electrode used as reference. Propofol and remifentanil were infused using a TCI system (FreseniusVial; Chemin de Fer, Béziers, France). All information *Ce*_*Prop*_, *Ce*_*Remi*_, BIS, RSS and GAG were annotated with a resolution of 1 second.

### EEG Preprocessing

The traditional bands analysis was performed on EEG signals filtered between 0.1–45 Hz and resampled at 128 Hz, while high frequency analysis was performed on EEG filtered between 0.1–145 Hz and resampled at 300 Hz. After the resampling process, the EEG signals were segmented in windows of length of 1 minute taken between 30 s and 90 s before the response annotation of RSS or GAG, in order to avoid the effect of the stimulation on the signal. The selected windows were filtered into the following frequency bands: *δ*, 0.1–4 Hz; *θ*, 4–8 Hz; *α*, 8–12 Hz; *β*, 12–30 Hz; *HF*; 60–95 Hz and *VHF*; 105–145 Hz; *TB*, 0.1–145 Hz. The frequencies round 50 Hz and 100 Hz were not taken into account in order to avoid the power line noise and the interferences caused by the self-test impedance device that produce a peak in the spectrum. Finite impulse response (FIR) filter of 50^th^ order was used in the present work. The order of the FIR filter was fixed to 50 in order to ensure the attenuation and the ripple in the stop band to be less than 5%.

The annotated RSS was assigned to the previous 1 minute length window if the differences *ΔCe*_*Remi*_ and *ΔCe*_*Prop*_ between the first and the last second of the window were *ΔCe*_*Remi*_<0.1 ng/ml and *ΔCe*_*Prop*_<0.1 μg/ml. Otherwise, the window was taken till the sample where the conditions were satisfied.

Firstly, a visual analysis was performed in order to eliminate EEG recording affected by strong noise of different types. Then, windows of EEG containing high amplitude peak noise were processed with a filter based on the analytic signal envelope (ASEF) [21]. This permitted to eliminate the peak noise, preserving the EEG frequency content. The FIR filter permitted to eliminate noise that have different frequency band from EEG signal. Another algorithm were applied in order to detect if adjacent samples differ more than 10% of the mean of the previous samples differences. In that case, those samples and the subsequent ones were not taken into account. In this way, the smallest analyzed window resulted to be of 50 s.

### Traditional EEG analysis

The following measures were calculated in each EEG window:

- Standard deviation (*std*) of the EEG windows filtered in each band.
- Power spectral density (PSD) for each EEG window in *TB* band using the Welch method.
- Spectral power in each band (*P*_*δ*_, *P*_*θ*_, *P*_*α*_, *P*_*β*_, *P*_*HF*_, *P*_*VHF*_) as the area under the PSD curve normalized by the total PSD area.
- Weighted mean frequency (*mF*) in each band (TB, *δ*, *θ*, *α*, *β*, *HF*, *VHF*) as the centroid of the PSD curve.
- Spectral edge frequencies *SEF*50, *SEF*75 and *SEF*90 in each band.
- Autocorrelation function (Ac)

The PSD was computed using Welch’s method of averaged modified periodograms. EEG segments were divided into one-second segments with 25% of overlap; Hamming window was applied to each segment. The final spectral density was achieved as the average of spectral densities of all the segments.

### Auto-Mutual Information Measures

Mutual information (MI) can measure the nonlinear as well as linear dependence of two variables. It is a metric derived from Shannon’s information theory to estimate the information gained from observations of one random event on another [22,23]. Usually, MI is measured between two different systems *X* and *Y*. Let *X* and *Y* be discrete random variables which take a finite number of possible values *x*_*i*_ and *y*_*j*_ with probabilities *P*_*x*_(*x*_*i*_) and *P*_*y*_(*y*_*j*_) respectively, ≥ 0, *i =* 1, 2, 3*…n* and *j =* 1, 2, 3*…n*, MI between *X* and *Y* is given by [22,23]

$$MI_{xy}=\underset{ij}{\sum }P_{xy}\left(x_{i},y_{j}\right){\text{log}}_{2}\left(\frac{P_{xy}\left(x_{i},y_{j}\right)}{P_{x}\left(x_{i}\right)P_{y}\left(y_{j}\right)}\right)$$ (1)

Auto-mutual information function (AMIF) (2) is calculated as the MI between two measurements *x*_*i*_ and *x*_*i+τ*_ taken from a single time series.

$$AMIF\_Sh(\tau )=\underset{x_{i}\epsilon X}{\sum }\underset{x_{i+\tau }\epsilon X}{\sum }P_{xx}\left(x_{i},x_{i+\tau }\right){\text{log}}_{2}\left(\frac{P_{xx}\left(x_{i},x_{i+\tau }\right)}{P_{x}\left(x_{i}\right)P_{x}\left(x_{i+\tau }\right)}\right)$$ (2)

AMIF function describes how the information of a signal (*AMIF* value at *τ* = 0) decreases over a prediction time interval (*AMIF* values *τ*>0). Increasing information loss is related to decreasing predictability, and increasing complexity of the signal [24].

AMIF can be also defined from Rényi entropy as

$$AMIF\_Re_{q}\left(\tau \right)=\frac{1}{q-1}log_{2}\underset{x_{i}\epsilon X}{\sum }\underset{x_{i+\tau }\epsilon X}{\sum }\frac{P_{xx}{}^{q}\left(x_{i},x_{i+\tau }\right)}{P_{x}{}^{q-1}\left(x_{i}\right)P_{x}^{q-1}\left(x_{i+\tau }\right)}$$ (3)

where *q* is the control parameter of Rényi entropy.

In Eq (3), the largest probabilities most influence the *AMIF_Re*_*q*_ when *q*>1 and the smallest probabilities most influence the values of *AMIF_Re*_*q*_ when 0<*q*<1. The *AMIF_Re*_*q*_ converge to the Shannon AMIF when *q* → 1. In this work, different values of the control parameter of *Re*: *q* = {0.1, 0.2, 0.5, 2, 3, 5, 10, 30, 50, 100} were taken into account.

The probabilities *P*_*xy*_, *P*_*x*_ and *P*_*y*_ were constructed on the series *x*_*i*_ or *y*_*j*_ and their delayed series *x*_*i+τ*_ or *y*_*j+τ*_ for *τ* = {1,2,…,300} samples. The amplitude range of the data series was quantized using 32 equidistant partitions. This made the maximum possible value of AMIF equal to log_2_ 32 = 5 bits. The number of bins was fixed following the recommendation of a previous work [25] which demonstrated that AMIF indexes applied to EEG were more stable and showed the less variability using 32 partitions. The τ limit was fixed to 300 samples that correspond to 1 s in order to find a compromise between the considered EEG frequency bands, the non-stationary characteristics of the EEG and the computing time. In this way, also the lowest frequency band (*δ* = 0.1–4 Hz) could be explored with a significant sample resolution (*τ*_*δ*_> *fs*/ *f*_*δ*_, *fs* = 300 Hz, *f*_*δ*_ <4 Hz: *τ*_*δ*_ >75 samples). Furthermore, the assumption of the EEG signal stationarity is ensured in the time delay of *τ* = 1 s and a reasonable computational cost is guaranteed.

The *q* range was chosen in order to have a distribution of *q*<1, *q* = 1 and *q*>1 values. Previous studies [26,18] demonstrated that AMIF has significantly changes if those partitions of *q* were taken into account and that the statistical results did not change in a significantly way in the range 50>*q*>100. We recognize that it would be more interesting and useful to analyze a fine partition of *q* values. However, the optimization of *q* value for AMIF would involve further analysis that are out of the purpose of this study. This can be considered as a future step in order to optimize the performance of a clinical indexes based on AMIF.

AMIF was normalized by the maximum value (AMIF(0)).

### Data Analysis

The AMIF function was analyzed with respect to *τ* in order to define measures able to discriminate between RSS scores. Then, several tests were performed on the EEG measures focusing on the discrimination between no-response (RSS = 6), sluggish responses (RSS = 5) and stronger responses (RSS<5). Firstly, EEG windows associated with sluggish responses (RSS = 5) were compared with no-responses (RSS = 6) in order to find measures that can—distinguish between adjacent sedation levels, trial1. Then, EEG windows associated with unresponsive states (RSS = 6), were compared with windows associated to all the responsive states (RSS<6), trial2, in order to discriminate between unresponsive and all the responsive states. In the following RSS tests, the measures calculated on EEG windows associated with the stronger stimuli responses (RSS<5) were compared with sluggish responses (RSS = 5), trial3, then with no-responses (RSS = 6), trial4, and finally with both sluggish and no-responses (RSS≥5), trial5. These last trials might help to improve the discrimination between stronger responses associated with low sedation levels and sluggish and no-responses associated with deeper sedation levels. In all the trials the unresponsive state is caused by an interaction between hypnotic and analgesic effects, while the responsive states are characterized by an insufficient presence of analgesic effect in the drug combination administered, especially when 2≤RSS<5.

Finally, the proposed variables that gave the highest *Sen*, *Spe* and *Pk* in the RSS prediction were used in GAG assessment, in order to evaluate their applicability on the prediction of the response to a different type of stimulus.

### Statistical Analysis

The U of Mann-Whitney test, was applied and a significance level p-value <0.05 was taken into account. Bonferroni correction was applied in case of multivariate comparison. Measures that showed statistical significance were considered for building univariable and multivariable discriminant functions.

The leaving-one-out method was performed as validation. Sensitivity (*Sen*) was used to measure the ratio between correctly classified windows associated with pain responses (RSS<6 and GAG 1) and the whole number of windows associated with pain responses; while specificity (*Spe*) was used to measure the ratio between correctly classified windows associated with no response (RSS = 6 and GAG 0) and the whole number of windows associated with no response.

The ability of the variables to describe pain responses was also evaluated using prediction probability (*Pk*), a statistic commonly used to compare the performance of indicators [27]. The standard error (SE) of the estimated *Pk* was also calculated in order to assess its accuracy. For the comparison between the *Pk* values obtained from the BIS and the EEG measures, a T-Student with significance level of 5% was performed. In order to build the multivariate model, an algorithm of feature selection was developed and applied to all the calculated measures that showed statistical significance (p-value<0.05) in at least one of the trial. The algorithm randomly selects a maximum of 4 not correlated measures and performed the multivariable discriminant analysis. More than 1000 iterations of the algorithm were performed in order to maximize the *Pk* under the condition of both Sen and Spe >60%. The best obtained combination are shown in the results section.

## Results

**Fig 1:**
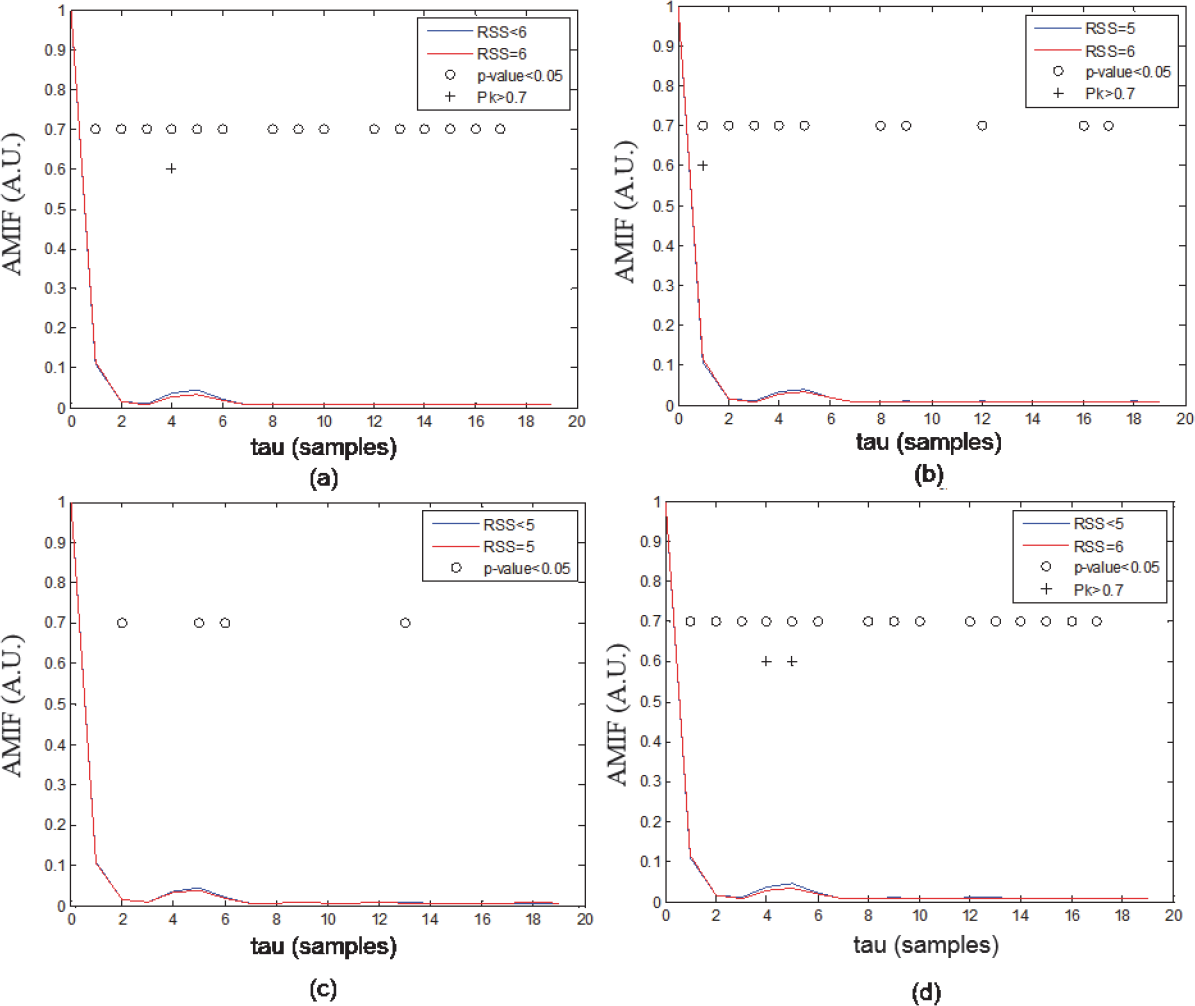
Auto-Mutual Information Function (AMIF). Averaged AMIF functions in VHF (105–145 Hz) band q = 0.5 of all windows of (a) RSS<6 and RSS = 6, (b) RSS = 5 and RSS = 6, (c) RSS<5 and RSS = 5, (d) RSS<5 and RSS = 6. The p-values were corrected using Bonferroni equation.

**Fig 2:**
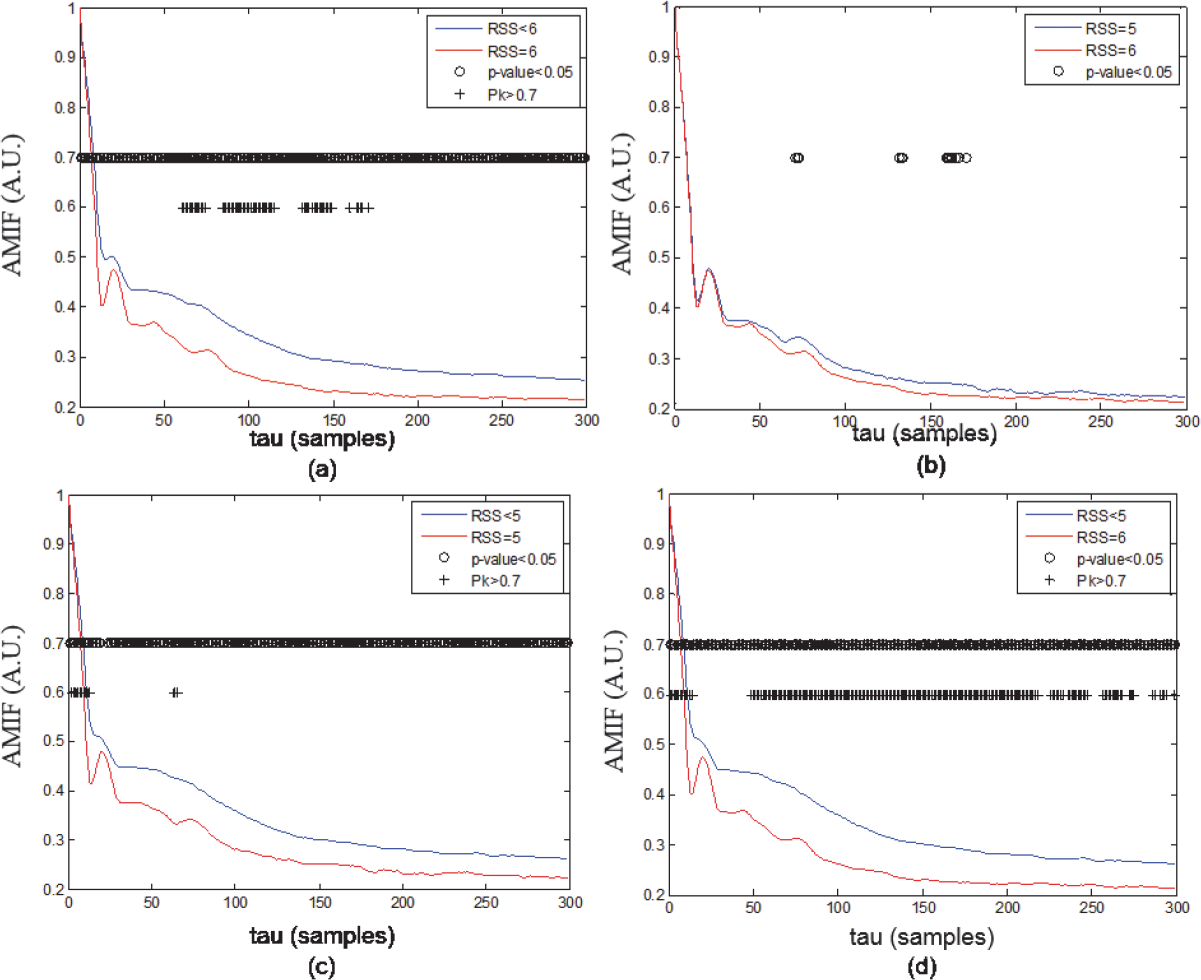
Auto-Mutual Information Function (AMIF). Averaged AMIF functions in *θ* band q = 50 of all windows of (a) RSS<6 and RSS = 6, (b) RSS = 5 and RSS = 6, (c) RSS<5 and RSS = 5, (d) RSS<5 and RSS = 6. The p-values were corrected using Bonferroni equation.

**Fig 3:**
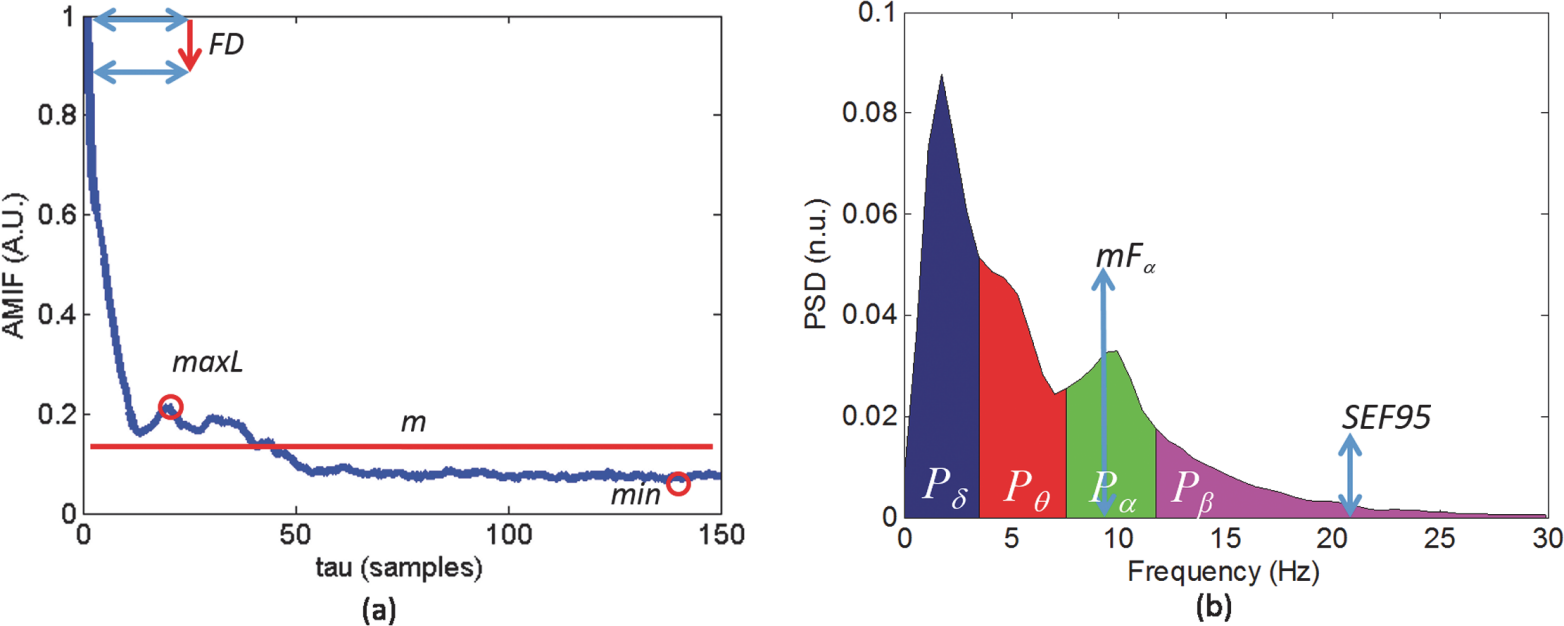
Measures extracted from Auto-Mutual Information Function (AMIF) and Power spectral density (PSD). Example of the measure calculation from (a) AMIF and (b) PSD.

As examples, Figs 1 and 2 show the averaged functions *AMIF_Re*_*05*_(*τ*) in *VHF* band and *AMIF_Re*_*50*_(*τ*) in *θ* band for different RSS levels, these two functions gave the most significant results in this first analysis. The *τ* delays in which those functions were able to yield p-value <0.05 and *Pk*>0.7 when discriminating between RSS<6 and RSS = 6, between RSS = 5 and RSS = 6, between RSS<5 and RSS = 5 and between RSS<5 and RSS = 6 are also represented. As it can be noted from Fig 1 and Fig 2, AMIF profiles exhibited an initial fast decrease at short time scale followed by a slow increase, then they decreased to nonzero stable values at longer time scales. Comparing RSS<6 and RSS = 6, few differences at short time-scale can be observed in *VHF* band (Fig 1A and Fig 1B) and at long time-scale in *θ* band (Fig 2A and Fig 2B), denoting different complexity behavior. Several statistical differences between RSS<5 and RSS = 5 or RSS = 6 are observed in *θ* band (Fig 2C and Fig 2D) both at short and long time-scales. From this first analysis it was deduced that the most appropriate measures with respect to delay *τ* that might quantify and extract the essential information contained on AMIF were: mean (*m*), first relative maximum (*maxL*), absolute minimum (*min*), and first decay (*FD*). The *FD* measure was calculated as the difference between the AMIF(0) and the AMIF(1). In this way, the values of the AMIF measures describe the complexity of the EEG signal: higher values of *m*, *maxL* and *min* are associated with less complexity, more regularity of the signal; contrarily higher values of *FD* are associated with more complexity and more irregularity of the signal. The same measures applied to *AMIF* were calculated on *Ac*. The EEG measures calculated on AMIF and PSD are summarized in Fig 3.

**Fig 4:**
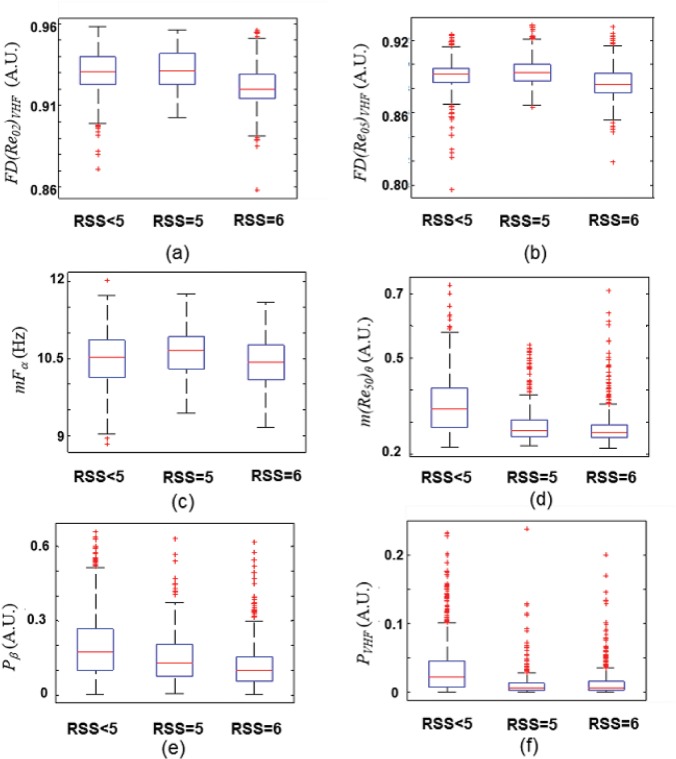
Values of EEG Measures with Ramsey score (RSS). Distribution of the values of (a) first decay of Auto-Mutual Information Function (AMIF) in VHF (105–145 Hz) band for q = 0.2, (b) first decay of AMIF in VHF (105–145 Hz) band for q = 0.5, (c) weighted mean frequency in *α* band, (d) mean value of AMIF in *θ* band for q = 50, (e) power spectral density in *β* band, (f) power spectral density in VHF (105–145 Hz) band. On each box, the central mark is the median; the edges of the box are the 25th and 75th percentiles. The whiskers are lines extending from each end of the boxes to show the extent of the rest of the data. Values beyond the end of the whiskers are considered outliers and marked with a +.

Fig 4 shows the values of the measures that gave the highest *Sen*, *Spe* and *Pk* in both univariable and multivariable discriminant functions.

From Fig 4 it can be noticed that when smallest probabilities predominate (*q* = 0.2, *q* = 0.5), RSS = 6 presents less complexity behavior than RSS = 5 in *VHF* (Fig 4A and 4B). The weighted mean frequency in *α* band is lower for RSS = 6 (Fig 4D) than RSS = 5. Furthermore, it can be observed that EEG windows of RSS<5 present less complexity behavior in *θ* band and higher power in *β* band than windows of RSS = 5 and RSS = 6.

Table 2 shows the results of the measures that gave the highest *Sen*, *Spe* and *Pk* using univariate discriminant function in trial 1 and trial 2. It can be noted that measures of AMIF in *VHF* are able to discriminate between RSS = 5 and RSS = 6 with *Sen*, *Spe* and *Pk* higher than *BIS* (T-student test, p-value<0.0001). However, only measures in *VHF* band permitted to discriminate between RSS = 5 and RSS = 6 with *Pk*>0.7.

**Table 2:** RSS assessment: trial 1 and 2.

	RSS = 5 vs RSS = 6 (N1 = 297, N2 = 701)			RSS<5 vs RSS = 6 (N1 = 1455, N2 = 701)		
Variables	*P*_*K*_	Sen	Spe	*P*_*K*_	Sen	Spe
*BIS*	0.620	63.8	56.3	0.799	75.5	68.6
*FD(Re*_*02*_ *)*_*VHF*_	0.719	61.6	70.0	0.715	64.4	69.5
*FD(Re*_*05*_ *)*_*VHF*_	0.709	62.0	68.0	0.678	65.0	63.6
*maxL(Re*_*2*_ *)*_*VHF*_	0.672	65.3	63.1	0.743	67.1	70.8
*m(Re*_*30*_ *)*_*δ*_	0.561	46.5	65.3	0.738	60.7	78.5

Uni-variable discrimination between EEG windows taken 30 s before nociceptive stimulation: response (RSS = 5 and RSS<6) and no-response (RSS = 6). *N1 = number of analyzed windows RSS<6; N2 = number of analyzed windows RSS = 6; P*_*k*_: prediction probability; Sen: (%) sensitivity; Spe: (%) specificity; p-value<0.05; Pk standard error: S.E. (Pk) < 0.02.

Table 3 shows the results of the measures that gave the highest *Sen*, *Spe* and *Pk* using multivariate discriminant function in trial 1 and trial 2. The combination of four measures permitted to yield *Sen*, *Spe* and *Pk* higher than *BIS* (T-student test, p-value<0.01) when discriminating between RSS<6 and RSS = 6. Table 4 shows the results of the measures that gave the highest *Sen*, *Spe* and *Pk* using multivariable discriminant function in trial 3, 4 and 5.

**Table 3:** RSS assessment: trial 1 and 2.

	RSS = 5 vs RSS = 6 (N1 = 297, N2 = 701)			RSS<5 vs RSS = 6 (N1 = 1455, N2 = 701)		
Variables *f(•)*	*P*_*K*_	Sen	Spe	*P*_*K*_	Sen	Spe
*mF*_*α*_, *FD(Re*_*05*_ *)*_*VHF*_, *maxL(Re*_*2*_ *)*_*HF*_, *min(Re*_*30*_ *)*_*δ*_	0.7633	70.7	70.5	0.8095	74.0	73.2
*maxL(Re*_*2*_ *)*_*VHF*,_ *m(Re*_*30*_ *)*_*δ*_, *m(Re*_*05*_ *)*_*α*,_ *maxL(Re*_*2*_ *)*_*θ*_	0.7036	63.0	65.5	0.8276	71.2	80.6
*FD(Re*_*2*_ *)*_*δ*_, *P*_*θ*_, *P*_*α*_, *maxL(Re*_*10*_ *)*_*α*_	0.6205	56.2	60.7	0.7555	60.1	78.9
*m(Re*_*50*_ *)*_*θ*_, *P*_*β*_, *P*_*VHF*_, *FD(Re*_*05*_ *)*_*VHF*_	0.7585	70.0	69.8	0.8338	72.2	79.3

Multi-variables discrimination between EEG windows taken 30 s before nociceptive stimulation: response (RSS = 5 and RSS<6) and no-response (RSS = 6). *N1 = number of analyzed windows RSS<6; N2 = number of analyzed windows RSS = 6; P*_*k*_: prediction probability; Sen: (%) sensitivity; Spe: (%) specificity; U of Mann-Whitney test between RSS scores: p-value<0.05; Pk standard error: S.E. (Pk) < 0.02.

**Table 4:** RSS assessment: trial 3, trial 4 and trial 5.

	RSS<5 vs. RSS = 5 (N1 = 1158, N2 = 297)			RSS<5 vs. RSS = 6 (N1 = 1158, N2 = 701)			RSS<5 vs. RSS≥5 (N1 = 1158, N2 = 998)		
Variables	*P*_*K*_	Sen	Spe	*P*_*K*_	Sen	Spe	*P*_*K*_	Sen	Spe
*BIS*	0.7531	72.5	65.0	0.8381	78.5	71.0	0.8128	77.5	68.0
*m(Re*_*30*_ *)*_*δ*_, *maxL(Re*_*02*_ *)*_*α*_, *P*_*β*_, *FD(Sh)*_*β*_	0.8010	72.1	72.7	0.8590	77.3	78.5	0.8395	75.7	76.1
*m(Re*_*50*_ *)*_*θ*_, *P*_*β*_, *P*_*VHF*_, *FD(Re*_*05*_ *)*_*VHF*_	0.7680	66.3	75.1	0.8645	72.2	83.5	0.8303	69.3	81.9

Multi-variables discrimination between EEG windows taken 30 s before nociceptive stimulation: strong response (RSS<5) and sluggish (RSS = 5) and no-response (RSS = 6). *N1 = number of analyzed windows RSS<5; N2 = number of analyzed windows RSS≥5; P*_*k*_: prediction probability; Sen: (%) sensitivity; Spe: (%) specificity; U of Mann-Whitney test between RSS scores: p-value<0.05; Pk standard error: S.E. (Pk) < 0.02.

Table 5 shows the results of the prediction of GAG using the variables that gave the highest *Sen*, *Spe* and *Pk* in the previous trials. In general, combination of measures capable to discriminate between RSS<5 and RSS≥5 gave high discrimination performances in GAG assessment. The combination between *mF*_*α*_, *FD(Re*_*05*_ *)*_*VHF*_, *maxL(Re*_*2*_ *)*_*HF*,_ *min(Re*_*30*_ *)*_*δ*_ gave the highest performances in the discrimination between RSS = 5 and RSS = 6 (*Pk*> 0.75, *Sen* and *Spe* >70%) and yield *Pk*>0.8 in the discrimination between RSS<6 and RSS = 6. However, it was not able to predict the GAG responses (*Pk*<0.7). The combination between *m(Re*_*50*_ *)*_*θ*,_ *P*_*β*,_ *P*_*VHF*_, *FD(Re*_*05*_ *)*_*VHF*_ yielded *Pk>0*.*75* in all the trials and in the GAG prediction.

**Table 5:** GAG assessment: multi-variable discrimination between EEG windows taken 30 s before the tube insertion: absence (GAG = 0) and presence of gag reflex.

	GAG 0 vs. GAG 1 (N1 = 353, N2 = 107)		
Variables	*P*_*K*_	Sen	Spe
*BIS*	0.7118	61.9	71.1
*m(Re*_*30*_ *)*_*δ*_, *maxL(Re*_*02*_ *)*_*α*_, *P*_*β*,_ *FD(Sh)*_*β*_	0.7633	63.7	73.8
*FD(Re*_*2*_ *)*_*δ*_, *P*_*θ*_, *P*_*α*_, *maxL(Re*_*10*_ *)*_*α*_	0.8027	74.2	71.3
*m(Re*_*50*_ *)*_*θ*_, *P*_*β*_, *P*_*VHF*_, *FD(Re*_*05*_ *)*_*VHF*_	0.7542	68.2	72.0

*N1 = number of analyzed windows GAG = 1; N2 = number of analyzed windows GAG = 0; P*_*k*_: prediction probability; Sen: (%) sensitivity; Spe: (%) specificity; U of Mann-Whitney test between RSS scores: p-value<0.05; Pk standard error: S.E. (Pk) < 0.02.

**Fig 5:**
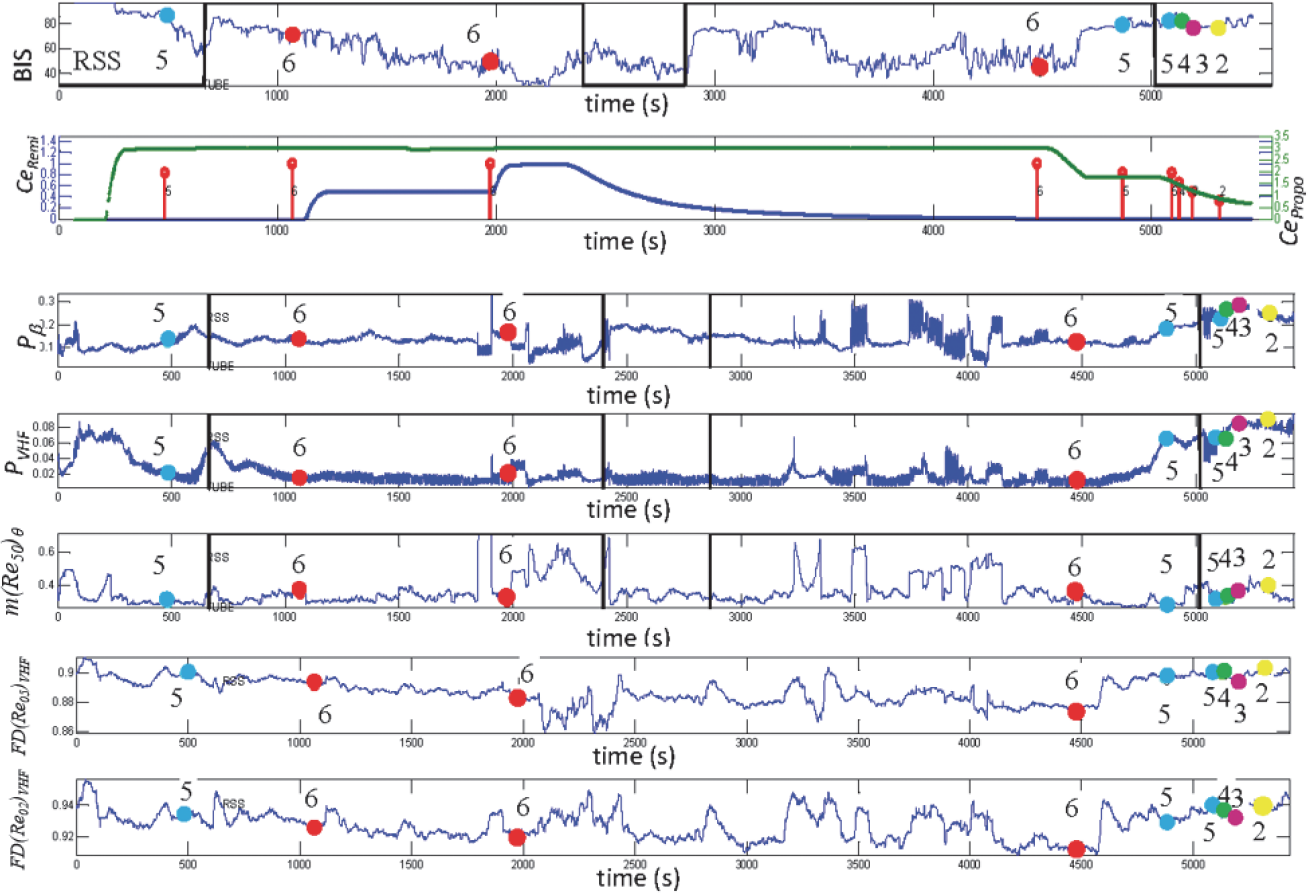
Time evolution of EEG measures. Example of time-evolution of bispectral index (BIS), predicted effect site remifentanil (Ce_Remi_ (ng/ml)) and propofol (Ce_Propo_ (μg/ml)), mean value of AMIF in *θ* band for q = 50, power spectral density in *β* band, power spectral density in *VHF* (105–145 Hz) band, first decay of Auto-Mutual Information Function (AMIF) in *VHF* (105–145 Hz) band for q = 0.5 and first decay of AMIF in *VHF* (105–145 Hz) band for q = 0.2 of a patient entire recording. All the EEG measures are represented in arbitrary units. Black vertical line represents the tube insertion, GAG = 1 in the first insertion and GAG = 0 in the second insertion. RSS annotation are represented with yellow (RSS = 2), pink (RSS = 3), purple (RSS = 4), blue (RSS = 5) and red (RSS = 6) circles.

As an example, Fig 5 shows the evolution of BIS, *Ce*_*Remi*_, *Ce*_*Propo*_, *m(Re*_*50*_ *)*_*θ*,_ *P*_*β*,_ *P*_*VHF*,_ *FD (Re*_*02*_ *)*_*VHF*_ and *FD (Re*_*05*_ *)*_*VHF*_ with respect to the entire recording time; the RSS annotation and the tube insertion are also shown. The EEG measures were calculated by sliding 60 s windows with steps of 1 s. As it can be noted, *P*_*β*,_ *P*_*VHF*_ *FD (Re*_*02*_ *)*_*VHF*_ and *FD(Re*_*05*_ *)*_*VHF*_ values are lower in the central part of the recording, when RSS = 6, while they increase when RSS<6. This implies less power and less complexity in high frequencies when the level of sedation is higher. The trend of *m(Re*_*50*_ *)*_*θ*_ denotes higher complexity in *θ* band for unresponsive state compared with RSS<5.

## Discussion

The proposed work reported a methodology for the prediction of pain responses during sedation based on EEG filtered in the traditional frequency bands and in higher frequency bands. In general, the combination of four measures in the traditional EEG bands is enough to yield *Pk*>0.8 when discriminating between RSS<5 and RSS≥5 (Table 4) and when predicting the gag reflex (Table 5). However, when discriminating between RSS<6 and RSS = 6 or between RSS = 5 and RSS = 6, only by using measures from EEG filtered at high frequency bands (*HF*; 60–95 Hz and *VHF*; 105–145 Hz) values of*Pk*>0.75 are obtained. In all the trials combinations of four EEG measures yielded discrimination performances better than BIS (T-student test, p-value<0.0001) in the prediction of pain responses. It is worth noting that the BIS index integrates several EEG measures such as time domain, frequency domain, and high order spectral parameters into a single variable. In this way, in order to make an appropriate comparison between the BIS and the results of the present study, the multi-variable analysis of EEG measures should be taken into account. However, it can be noted that, when discriminating between RSS = 5 and RSS = 6,BIS is outperformed by single measures calculated on EEG filtered in *VHF* (Table 2). It is well known that BIS is able to describe hypnotic effect as it is confirmed by results of trial 2, 4 and 5, since the responsive states of these trials is characterized by an insufficient presence of analgesic effect in the drug combination administered. In this case, the discrimination is performed between sedation levels characterized by different hypnotic states at low analgesic effect. However, as it was seen in trial 1, 3 and GAG the proposed multi variable functions showed a better capability than BIS to describe the analgesic effect. The presence of analgesic, remifentanil in this case, causes that the patient can not react to painful stimuli even with a low level of hypnotic agents (i.e. a high BIS value). If, instead of analgesic, only propofol were dispensed, its concentration would have to be higher in order to block painful stimuli and that would produce very low BIS values. The hardest challenge is the discrimination between sluggish response (RSS = 5) and no response (RSS = 6). In general, the major benefits in the discrimination of these two RSS levels were provided by studying changes in the power spectral density in *α* and *β* bands of EEG and in the complexity behavior of EEG filtered in high frequency band (60–95 Hz and 105–145 Hz). In order to yield high performances in the prediction of different stimuli responses, the complexity behavior of *δ* and *θ* bands and the power spectral density in *VHF* should also be taken into account.

In a previous work, spontaneous frontal electromyography (SEMG) has been shown to be a useful indicator of pending arousal [28]. Another study demonstrated that sudden increases in the amplitude of SEMG activity in frontal muscles during surgery indicated enhanced patient responsiveness [29]. This is due to the motor innervation of the upper facial muscles, which arises from the brainstem, with connections to vigilance centers in the reticular formation. Some successful applications of the SEMG on the detection of arousal during anaesthesia have been published [28–30]. However, due to great inter-individual variability in the amplitude of the SEMG, this technique has not gained wide clinical acceptance. In this work, the AMIF computation normalized by the maximum value permits to obtain non-linear measures that might limit the inter-individual variability.

Recently, Mathews et al. [31] showed that measures of electroencephalogram and EMG variability increased when intraoperative somatic events occurred and permitted to discriminate between 10-minute segments that contained a somatic event and those segments that did not contain an event. Their results have been demonstrated to be better than the methods based on changes in HR and mean arterial blood pressure. The contraction strength of a frontals muscle is linearly dependent on the firing rate of motoneurons, which innervate the frontals. As surface electrodes measure the sum of signals from multiple motor units, the work of Rautee *et al*. suggested that the complexity of the SEMG band is related to the level of desynchronization between firing rates among adjacent motor units. The results of spectral entropy in that study demonstrated that increased firing rates seem to produce increased desynchronization between motor units [32] associated with higher disorder of the system. In the present work, the EEG filtered in *VHF* bands presents less complexity in RSS = 6 than RSS≤5 being the value of the AMIF first decay lower (Fig 4A and 4B). In this way, the less complexity behavior of the EEG filtered at *VHF* might be associated with a decrease of the firing rates of the SEMG induced by the deeper level of sedation. This is corroborated by the lower values of the power in *VHF* observed in unresponsive states (Fig 4F) and in the central part of the time evolution in Fig 5.

Regarding the traditional EEG bands, previous studies have been demonstrated that increased sedation levels are marked by increased *δ* and *θ* power and frontal *α* with increased peak frequency [33–36]. Then, increasing propofol concentration in human subjects shifts cortical activity from a high-frequency, low-amplitude signal to a low-frequency, high-amplitude signal. Specifically, *β* activity decreases and *α* and *δ* activities increase [37] with increasing levels of propofol anesthesia. In Fig 4E it can be observed that *P*_*β*_ is lower for the unresponsive state reflecting a higher level of sedation respect unresponsive states. Also the weighted mean frequency in *α* band (Fig 4C) is lower for RSS = 6, this implies that the spectral power is higher in lower frequencies. In this work, patients in deep sedation showed a more complex EEG activity in low frequency bands than patients in lower sedation levels (Fig 4D). This can be related with the fact that EEG activity becomes slower as the sedation level increases, and thus, it is expected that patients in unresponsive state present a slower EEG signal than responsive state, increasing the signal complexity in low frequency bands. Power in the high-frequency component of EEG signals corresponds to corticocortical activity [37], while power in the low-frequency component of EEG signals primarily arises from subcortical interactions [38]. The decrease in high-frequency power with the administration of propofol may be due to a decrease in intracortical and corticocortical activity. The increase in low-frequency power may arise from interactions with subcortical structures such as the thalamus [36, 39]. EMG activity reflects subcortical activity during general anaesthesia. According to previous studies, subcortical structures could be a site of the analgesic effect of anaesthetics [40–42].

Most studies addressing mechanisms of sedation and consciousness have been performed under the effects of one drug only being it propofol or sevoflurane. Addition of an opioid as in this case might probably affect the mechanisms of sedation in several ways but by the analysis of the EEG collected in our study it is not possible to establish a mechanistic hypothesis other than the already demonstrated synergy between opioids and hypnotic drugs.

To our knowledge, there are not many publications addressing this topic of sedation-analgesia under combined propofol and remifentanil in real patients. One of them [43] have different RSS score target (RSS = 4) and another one only took into account the drug concentration for studying the loss of response [44] in volunteers undergoing a process similar to gastrointestinal endoscopy.

In previous studies, all the EEG derived indexes showed high statistical performances in the detection of the deep of anesthesia [45–46] with correlation scores or Pk >0.8.

However, general anesthesia requires deeper levels of hypnotic and opioid concentrations, most of the times with pharmacological muscular paralysis to allow for mechanical ventilation that eliminates any muscular activity improving the ability to predict certain responses with the EEG because it is free of EMG signal.

During sedation and analgesia patients must be comfortable, painless but breathing spontaneously. This means that there will be muscular activity. EEG derived measure such as BIS will perform worse in this context as demonstrated by the results of the present work. A measure that would take into account EMG activity by expanding the frequency spectrum to be analyzed, as proposed in the present investigation, would probably be a better candidate to predict sedation states better.

Auto-Mutual information function was chosen as a non-linear technique in order to evaluate the complexity behavior of the EEG filtered in the different bands. The fundamental assumption of nonlinear techniques is that EEG signal is generated by nonlinear deterministic processes with nonlinear coupling interactions between neuronal populations [47, 48]. In general, the most applied complexity measures found in the literature are attractor dimension [49], correlation dimension [50], Lyapunov exponent [51], fractal dimension [52], entropies [52], recurrence quantification analysis [53], higher order statistic and spectral [54]. Most of the complexity measures mentioned above depend on the setting of estimation parameters, namely embedding dimension, time delay of phase space reconstruction, prediction time horizon, and partitioning signals. Assuming that the dimensional complexity of the nonlinear system that generates the EEG signals is expected to be between 5 and 10 [55, 56], depending on the aware or unaware states, the limit of the time series length was defined between 10^3^ and 10^5^ samples [57] or even greater than 10^5^ [58]. These suppose a large sample size of the data while mutual information function that describes the amount of information of a signal with regard to a time shifted quantity can be constructed on short time series. This allows the AMIF data to be computed more frequently so that the overall diagnostic indicator can quickly indicate changes in the state of the patient. On the other hand, in this work mutual information requires the estimation of probabilities from limited EEG time series and this might affect the estimation of mutual information functions. However, in a previous work [59] this function was calculated using 1024 samples after demonstrating a stabilization of the mutual information measures variability for at least 512 samples. Furthermore, in other studies mutual information functions were estimated on EEG and heart beat time series with length between 10^3^ and 10^4^ samples [60, 61]. In the proposed work the smallest EEG window resulted to be of 50 s corresponding to 6400 samples at a sampling frequency of 128 Hz.

Furthermore, in the last decades, various time-frequency analysis methods have been proposed for analyzing, interpreting, and scoring EEG signals, such as the wavelet transform [62], the Hilbert-Huang transform (HHT) [63]. However, the wavelet transform requires the proper choice of a mother wavelet function and the HHT, which is based on empirical mode decomposition (EMD) [64], is very sensitive to the instantaneous energy content of the harmonics and depends on the chosen spline-fitting techniques and stopping criterion [65]. For this reason, the results might be perturbed [66], especially if random noise is present in the signal. In this way, before applying these methods on the present dataset further specific studies should be performed but that would be out of the purpose of this work. Furthermore, in the frequency representation provided by the wavelet transform and the HHT, there is no direct correspondence between the scale and wavelength or frequency. Also, the conversion factors that yield an approximate scale-frequency correspondence must be found. Regarding high order spectral, since this feature is already included in the bispectral index we decided to not apply it in the present study.

In this way, time-frequency representation based on Wigner-Ville and Choi-Williams distribution might be suitable for the purpose of this work for its ability to represent the spectral features at each time-instant. However, comparing the results of two previous works, applied to the same database, it can be noted that the statistical performances of the measures based on time-frequency representation [17] does not significantly improve the statistical performances of measures based on the Welch method [18]. In this way, since the Welch method avoids excessive computational charges and requires less computing time, it was considered to better fit with the purpose of this study.

In all the trials, indexes based on AMIF outperformed all the autocorrelation and spectral indexes in term of *Sen*, *Spe* and *Pk*. Especially, in the trial 1, only AMIF measures showed *Pk*>0.65, in EMG band. We assumed that the properties of AMIF to deal with non-linear dynamics permit to detect differences in complexity behavior between the EEG recorded in different sedation levels. One can say that, in the case of the present study, AMIF should not provide additional information because of the slightly presence of nonlinear dynamics in EEG signal in healthy people compared with people suffering relevant neural pathology. However, previous studies suggested the importance of nonlinear EEG analysis as a tool to monitor anesthetic depth [67, 68], since they found correlation between EEG complexity indexes and depth of anesthesia and drug concentrations. Furthermore, we supposed that in EMG frequency bands, the presence of nonlinear dynamics might be stronger than in EEG. In this way, EEG filtered in EMG frequency bands might have different complexity behavior when comparing different values of Ramsey scores.

A previous study [69] assessed that nonstationarity affect frequency and information parameters, requiring stationarity of the EEG in the application of power spectral and AMIF. However, several works demonstrated that, during wakefulness, EEG can be considered stationary for short intervals (about 1–20 s) and with increasing anaesthetic level, the probability and duration of stationary EEG increases [70–74]. In the present study, the effect of non-stationarity was minimized by averaging the power spectral density calculated with Welch method using a moving window of 1 s. In this short interval, we can use the assumption of stationary EEG. Regarding the application of AMIF, a compromise has to be made between the minimum window length for estimating the probability density and EEG stationarity. To solve this issue, we applied AMIF to one-minute EEG windows and we took into account values of time delay *τ* lower than 1 s, in AMIF calculation. Furthermore, the EEG windows were taken into account only if no-variation higher than 0.1 ng/ml of remifentanil and 0.1 μg/ml of propofol were observed. In this way, we might assume that EEG did not have strong variations due to variation in the drug concentrations and thus the sedation level did not change. From all these considerations, we assume that, even though the analyzed window were of one-minute length, the non-stationarity effect on our results were minimized.

Analyzing all the results it can be denoted that the combinations of linear and non-linear measures calculated in the traditional EEG bands were able to yield *Pk*>0.8 when discriminating between RSS<5) and (RSS = 5 and/or RSS = 6) and between GAG 0 and GAG 1. However, in order to yield *Pk*>0.8 when discriminating between RSS<6 and RSS = 6, measures calculated in the traditional EEG bands had to be combined with measures calculated in EEG filtered at high frequencies where the SEMG component is present. In this way, the measures calculated in EEG filtered at high frequencies improved the prediction of unresponsive (RSS<6 and GAG = 0) and responsive (RSS = 6 and GAG = 1) states when different types of stimuli are taken into account.

The use of combination between linear and non-linear EEG measures gave the advantages to deal with both the spectrum and complexity characteristics of the EEG signal. Furthermore, by calculating EEG filtered in high frequency bands, it could be possible to improve the pain responses prediction by including the contribution of the EMG component. In this way, further than the hypnotic effects, also the analgesic effects was better characterized. This can give a strong improvement in the prediction of the response to noxious stimuli in patients under anesthesia and sedation leading to design a method which can be integrated into a medical device for the assessment of pain and nociception during anaesthesia. It was demonstrated that intraoperative monitoring of processed physiological signals reduced anesthetic exposure improving early recovery profiles and faster emergence from anesthesia [75]. Furthermore, it decreased the risk of post-operative delirium and cognitive decline [76]. Hence the outcome will be of benefit for the patients and have a considerable socio-economic impact.

Since the nociceptive stimuli analyzed in the present work are of different kind, our findings might be extended in any kind of surgical procedures, where patients are sedated with both analgesic and hypnotic agents.

However, a limitation of this study can be the use of a database in which only two kind of anesthetics were used (propofol and remifentanil) because it represented a particular case of drug. For a further validation, this method should be also applied to patients undergoing sedation with different drug administrated (i e. sevoflurane)

## Conclusions

Measures extracted from EEG filtered at high-frequencies have been proposed as new approach to monitor pain responses during sedation. Traditional measures based on power spectral density and nonlinear measures based on auto-mutual information function (AMIF) applied to EEG filtered in the traditional bands and in high frequency bands improved the discrimination between different sedation levels. Single variables based on nonlinear measures of EEG filtered in *VHF* band (105–145 Hz) yielded *Pk* >0.7, *Sen*>60 and *Spe*>65 when predicting Ramsey sedation score (RSS) related to between nociceptive responses RSS = 5 and non-responses RSS = 6 to nail bed compression.

The combination of variables based on the spectral power in *β* and *VHF* bands, the mean value of the AMIF calculated with q>1 in *θ* band and the first decay of AMIF calculated with q<1 in *VHF* (105–145 Hz) band yielded *Pk>0*.*75* in all RSS and GAG prediction.

The obtained results indicate that measures from frontal EEG filtered in high frequencies that contain also EMG components helped to improve the prediction of different stimuli responses. The combination of linear and non-linear measures of EEG filtered in the traditional bands and in higher frequencies resulted to be a promising methodology for the non-invasive prediction of pain responses during sedation. This might give a contribution to solve the problems related to the development of a clinical index able to assess the sedation level.

## Glossary

- *BIS:* Bispectral index
- *FD(Re_02_)_VHF_:* First decay (FD) of AMIF using Rényi entropy (Re) with control parameter q = 0.2 in the VHF frequency band
- *FD(Re_05_)_VHF_:* First decay (FD) of AMIF using Rényi entropy (Re) with control parameter q = 0.5 in the VHF frequency band
- *FD(Re_2_)_δ_:* First decay (FD) of AMIF using Rényi entropy (Re) with control parameter q = 2 in the δ frequency band
- *FD(Sh)_β_:* First decay (FD) of AMIF using Shannon entropy (Sh) in the _β_ frequency band
- *m(Re_05_)_α_ :* Mean (m) of AMIF using Rényi entropy (Re) with control parameter q = 0.5 in the α frequency band
- *m(Re_30_)_δ_:* Mean (m) of AMIF using Rényi entropy (Re) with control parameter q = 30 in the δ frequency band
- *m(Re_50_)_θ_*: Mean (m) of AMIF using Rényi entropy (Re) with control parameter q = 50 in the θ frequency band
- *maxL(Re_02_)_α_:* First relative maximum (maxL) of AMIF using Rényi entropy (Re) with control parameter q = 0.2 in the α frequency band
- *maxL(Re_10_)_α_:* First relative maximum (maxL) of AMIF using Rényi entropy (Re) with control parameter q = 10 in the α frequency band
- *maxL(Re_2_)_HF_*: First relative maximum (maxL) of AMIF using Rényi entropy (Re) with control parameter q = 2 in the HF frequency band
- *maxL(Re_2_)_VHF_:* First relative maximum (maxL) of AMIF using Rényi entropy (Re) with control parameter q = 2 in the VHF frequency band
- *maxL(Re_2_)_θ_*: First relative maximum (maxL) of AMIF using Rényi entropy (Re) with control parameter q = 2 in the θ frequency band
- *min2(Re_30_) _δ_:* Absolute minimum (min2) of AMIF using Rényi entropy (Re) with control parameter q = 30 in the δ frequency band
- *mF_α_:* Weighted mean frequency (mF) in α frequency band
- *P_α_:* Spectral power in α frequency band
- *P_β_:* Spectral power in β frequency band
- *P_VHF_:* Spectral power in VHF frequency band
- *P_θ_:* Spectral power in θ frequency band
